# The Murine Gammaherpesvirus-68 gp150 Acts as an Immunogenic Decoy to Limit Virion Neutralization

**DOI:** 10.1371/journal.pone.0000705

**Published:** 2007-08-08

**Authors:** Laurent Gillet, Janet S. May, Susanna Colaco, Philip G. Stevenson

**Affiliations:** Division of Virology, Department of Pathology, University of Cambridge, Cambridge, United Kingdom; University of California at San Francisco, United States of America

## Abstract

Herpesviruses maintain long-term infectivity without marked antigenic variation. They must therefore evade neutralization by other means. Immune sera block murine gammaherpesvirus-68 (MHV-68) infection of fibroblasts, but fail to block and even enhance its infection of IgG Fc receptor-bearing cells, suggesting that the antibody response to infection is actually poor at ablating virion infectivity completely. Here we analyzed this effect further by quantitating the glycoprotein-specific antibody response of MHV-68 carrier mice. Gp150 was much the commonest glycoprotein target and played a predominant role in driving Fc receptor-dependent infection: when gp150-specific antibodies were boosted, Fc receptor-dependent infection increased; and when gp150-specific antibodies were removed, Fc receptor-dependent infection was largely lost. Neither gp150-specific monoclonal antibodies nor gp150-specific polyclonal sera gave significant virion neutralization. Gp150 therefore acts as an immunogenic decoy, distorting the MHV-68-specific antibody response to promote Fc receptor-dependent infection and so compromise virion neutralization. This immune evasion mechanism may be common to many non-essential herpesvirus glycoproteins.

## Introduction

Antibody is a potent force in anti-viral immunity, neutralizing virions [1] and promoting infected cell lysis [2]. But while antibody renders many epidemic viruses non-infectious, it fails to stop the transmission of persistent viruses. Herpesviruses provide a paradigm. They both enter and exit immune hosts in the presence of antibody [3], and moreover show little antigenic variation in so doing [4], suggesting that antibody even fails to impose much selective pressure. Poor neutralization within hosts is easy to understand: herpesviruses spread mainly through cell/cell contacts, which can exclude antibody [5]. Thus, the pseudorabiesvirus gD, which is required for virion entry, is dispensible for dissemination in vivo [6], while the Herpes simplex virus gE/gI complex, which contributes to cell/cell spread, is important [7]. In this setting, antibody must act mainly through cytotoxicity [8], and alpha- and beta-herpesvirus consequently encode receptors that blunt Fc-dependent attack [9], [10]. Gamma-herpesviruses do not, but their host colonization relies more on latency-associated lymphoproliferation than on lytic replication [11]–[13].

Poor neutralization of the cell-free virions that transmit infection between hosts is more difficult to understand. Antibody is clearly capable of neutralizing mucosal virions in other infections [14]. Also, herpesviruses presumably operate in antibody excess at the steady state of persistent infection-any antigen excess would simply elicit more antibody. Alpha-herpesviruses may overwhelm pre-formed antibody by intermittent, large scale reactivations, and by replicating in the epidermis where antibody is sparse [15]. But gammaherpesvirus shed virions fairly constantly and in mucosal sites where antibody is abundant [16]. Thus, the argument of quantitative antibody deficiency seems for gamma-herpesviruses to be unconvincing. However, poor gammaherpesvirus neutralization might reflect qualitatively sub-optimal antibody responses. Despite much functional analysis of whole antibody responses and many examples of viral glycoprotein-specific monoclonal antibodies (mAbs), there has been little break-down of how well gammaherpesvirus carriers target each virion glycoprotein. This is important: fewer than half of the the virion glycoproteins are essential, and it is far from clear that antibody targets these selectively. Even an essential glycoprotein will not be a good neutralization target unless it is immunogenic in natural infection.

Our understanding of gammaherpesvirus neutralization has been limited in part by the narrow species tropisms of Epstein-Barr virus (EBV) and the Kaposi's Sarcoma-associated Herpesvirus (KSHV). The major neutralization target defined for EBV-gp350 [17]-is dispensible for epithelial infection [18]. It would anyway seem unlikely that gp350-specific antibodies could stop EBV transmission [19]. Neutralization targets apart from gp350 exist [20], but cognate antibodies may be rare. The difficulties of analyzing EBV and KSHV directly have made related viruses an important source of information. One of the most experimentally accessible is murine gammaherpesvirus-68 (MHV-68), a natural, B cell-tropic parasite of mice [21]–[23]. There are limitations on MHV-68 analysis too-for example, a transmission model has not yet been developed. Nevertheless, it allows a ready quantitation of antibody responses and experimental infection with wild-type and targetted mutant viruses. Immune sera inhibit MHV-68 infection of fibroblasts [24], probably by blocking cell binding [25]. The limitations on this mode of neutralization are apparent when virus/antibody complexes meet IgG Fc receptors: immune sera then fail to block infection [26], indicating that viral membrane fusion still works. What does the MHV-68-specific antibody response target? The major monoclonal antibody (mAb)-defined neutralization target is gH/gL [25]. Here we show that the response mounted against the accessible virion surface is dominated not by gH/gL-a comparatively minor target-but by the non-essential gp150. Gp150 was not a significant neutralization target and instead accounted for much of the capacity of immune sera to drive IgG Fc receptor-dependent infection. Antibody immunodominance therefore worked against neutralization.

## Results

### Gp150 dominates immunological footprint of MHV-68 virions

We aimed to quantitate the antibody response of MHV-68 carriers to each accessible virion glycoprotein. A key task was to minimize detection bias. Assays based on recombinant protein expression potentially allow direct analyses of immune sera. However, each recombinant protein inevitably varies in how well it reproduces native virion epitopes. This creates bias. Also, recombinant proteins may display other epitopes that are accessible on disrupted virions and therefore immunogenic in vivo, but are not accessible on intact virions. Another problem with analyzing immune sera is that non-specific staining can make weak, specific staining hard to discern. We therefore based our approach on generating B cell hybridomas from MHV-68-infected mice. We made use of the fact that MHV-68-infected cells capture virions on their surfaces [27] and should therefore display all the virion glycoproteins in their native forms. Thus, we identified antibodies as potentially (but not necessarily) virion glycoprotein-specific if they recognized unfixed, MHV-68-infected BHK-21 cells. MAbs were selected simply for positive staining, without any discrimination towards stronger or weaker staining. Each was then typed for its glycoprotein target (Table 1) [28]–[34]. Although infected cells may obviously also display non-virion antigens, essentially all the mAbs that recognized infected cell surfaces were found to recognize a virion glycoprotein. Using the assays outlined in Table 1, we consistently identified targets for more than 95% of all infected cell surface-specific hybrids.

**Table 1:** Identification of MHV-68 glycoprotein-specific mAbs.

Protein	Primary identification	Secondary identification
gB [28]	transfected cell line	120kDa IP product
gH/gL [25]	85kDa IP product	transfected cell line
gp48 [29]	knockout virus	transfected cell line
ORF58 [30]	knockout virus	transfected cell line
gp70 [31]	transfected cell line	knockout virus
ORF74 [32]	knockout virus	transfected cell line
gp150 [27]	knockout virus	transfected cell line
ORF28 [33]	knockout virus	transfected cell line
gN/gM [34]	transfected cell line	35kDa IP product

**Figure 1:**
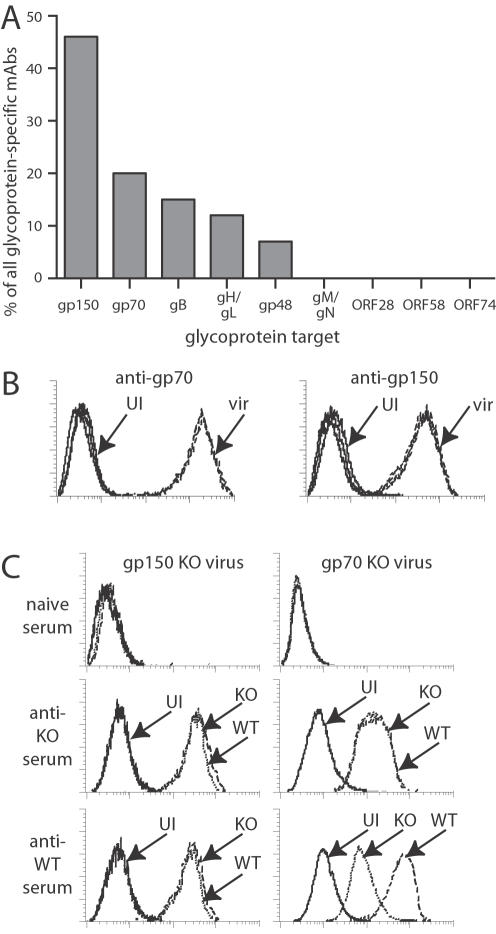
Gp150 is the most immunogenic MHV-68 glycoprotein. A. Hybridomas were derived from the pooled spleens of 3 BALB/c mice 5 months after intranasal MHV-68 infection. Each mAb that recognized unfixed, MHV-68-infected BHK-21 cells was typed for its glycoprotein target as outlined in Table 1. None remained unaccounted for. The data shown are from 1 fusion experiment that generated 128 glycoprotein-specific mAbs. 5 further experiments all gave similar results. B. BHK-21 cells were either uninfected (UI, solid lines) or infected with wild-type MHV-68 (2 PFU/cell, 18h: vir, dashed lines) and stained with mAbs specific for gp70 or gp150. Histograms for 3 mAbs, all used in saturating amounts, are overlaid in each graph. Gp70-specific staining was consistently stronger than that of other viral glycoproteins, including gp150. C. BHK-21 cells were either uninfected (UI, solid lines) or infected (2 PFU/cell, 18h) with wild-type (WT, dashed lines), gp150-deficient (gp150 KO, dotted lines) or gp70-deficient (gp70 KO, dotted lines) viruses. The cells were then stained with sera from mice infected with wild-type, gp150-deficient or gp70-deficient viruses, or from age-matched naive mice.

In initial experiments, we infected mice with latency-deficient MHV-68 [35] to avoid the risk of persistent virus contaminating the hybridomas. In subsequent experiments with wild-type virus (Fig. 1A), this was not found to be a significant problem. The frequencies of glycoprotein-specific mAbs obtained were very similar. Assuming that splenic B cells are representative of the whole B cell response and that B cell specificity does not bias hybrid formation, then splenic hybridoma frequency should reflect immunogenicity. By this measure, gp150 was much the most immunogenic virion glycoprotein. It was typically the target of 50% of all infected cell surface-specific mAbs. In one fusion it reached 69%. In the largest fusion analyzed, 94 (51%) of 184 mAbs recognizing infected cell surfaces recognized gp150. In the experiment shown (Fig. 1A), all infected cell surface-specific mAbs recognized either gp150, gp70, gB, gH/gL or gp48. In other fusions, we also identified mAbs against gN, but we failed to find any against ORF28 gene products or gM. These are probably hard for antibody to reach: gM is largely confined to the plane of the virion membrane, while the ORF28 protein is both small and heavily glycosylated [33]. ORF58 also encodes a virion component (unpublished data), but this associates with and is presumably protected by the heavily glycosylated gp48 [30]. The immunogenic virion surface therefore comprised gp150, gp70, gB, gH/gL, gp48 and gN. The only mAb-defined MHV-68 neutralization target apart from gH/gL is gB [36]. Thus, immunodominance did not correlate with neutralization; if anything, the converse was true. This was consistent with good neutralizing mAbs being quite rarely recovered from virus carriers and with the whole response blocking fusion poorly.

Gp150 immunodominance did not simply reflect abundant expression. Gp70-specific mAbs stained MHV-68-infected cells more strongly than gp150-specific mAbs (Fig. 1B), and while immune sera stained gp70-knockout-infected cells less well than wild-type, the staining of gp150-knockout-infected cells was the same (Fig. 1C). Gp70 was therefore more highly expressed than gp150, implying that gp150 was intrinsically more immunogenic.

### A relatively small region of gp150 accounts for much of its immunogenicity

**Figure 2:**
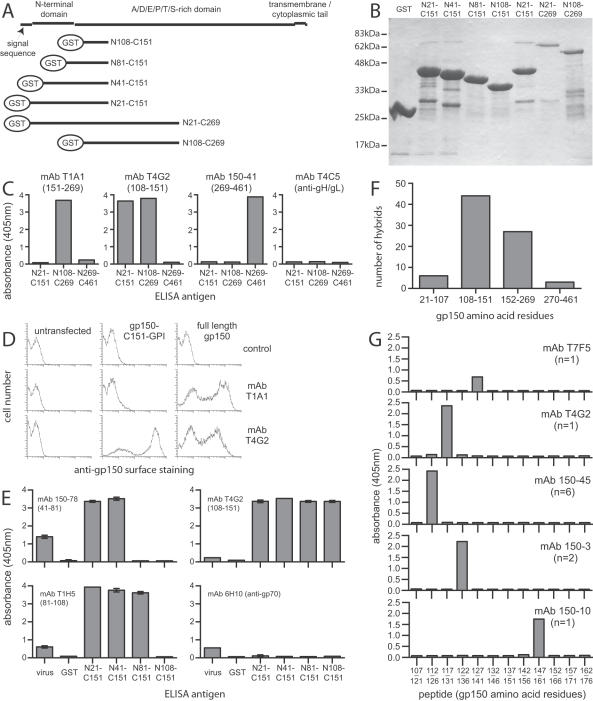
Epitope mapping for gp150-specific mAbs. A. Gp150 comprises an N-terminal signal sequence, a small N-terminal domain, a long stalk rich in anionic residues, proline residues and potential O-glycosylation sites (A/D/E/P/T/S-rich domain), a transmembrane domain and a short cytoplasmic tail. Our analysis focussed on the membrane-distal half of the protein. B. GST fusion proteins were purified with glutathione-sepharose, separated by SDS-PAGE and Coomassie stained. Longer regions of the stalk domain were less well expressed than shorter forms and were subject to more degradation. C. MAb recognition was mapped by ELISA with GST fusion proteins covering the gp150 extracellular domain. Examples of fusion protein recognition are shown (mAbs T1A1, T4G2 and 150-41, with their deduced recognition sites given below). Despite the limited amino acid diversity of gp150, little mAb cross-reactivity was evident between the expressed protein segments. The gH/gL-specific mAb T4C5 provided a negative control. D. Recognition patterns were confirmed by flow cytometric staining of 293T cells transfected with either full-length gp150 or the N-terminal 151 residues of gp150 with a GPI membrane anchor. Examples of staining are shown. Control = secondary antibody only. E. More detailed analysis of the N21-C151 region, which accounted for most gp150-specific mAbs, using N-terminal gp150 truncations. Virus = wells coated with 0.01% Triton X-100-disrupted MHV-68 virions, GST = GST alone. Examples of recognition patterns are shown. The gp70-specific mAb 6H10 provided a negative control for gp150 recognition. F. Summary of GST fusion protein recognition by 81 gp150-specific mAbs. G. MAb specificities within the 108-151 region were mapped with overlapping 15-mer biotinylated peptides bound to avidin-coated plates. The amino acid residues covered by each peptide are indicated. Examples of recognition patterns are shown with the total number of mAbs showing that pattern.

The 460 amino acid extracellular domain of gp150 is predicted to be strongly anionic with>100 O-linked glycosylation sites. Discounting the predicted signal peptide (residues1–22) and a small N-terminal domain (residues23–93), 94% of its amino acids are A, D, E, P, S or T. The bulk of gp150 is therefore likely to form an extended, hydrophilic stalk stabilized by O-linked glycans. It readily binds to the O-glycan-specific lectin jacalin [33]. We identified particularly immunogenic regions of gp150 by ELISA of protein fragments fused to GST (Fig. 2A, 2B). MAbs were first tested against the entire gp150 extracellular domain in 3 segments: residues 21–151, 108–269 and 269–461 (Fig. 2C). These results were confirmed by testing reactivity against cells transfected with either full-length gp150 or a truncated form comprising its first 151 residues plus a glycosyl-phosphatidylinositol membrane anchor (Fig. 2D). Most mAbs recognized the region up to residue 151. We mapped recognition more finely within residues 21–151 using a series of N-terminal truncation mutants (Fig. 2E). No mAbs recognized residues 21–41 and few recognized 42–107. A relatively small region of the gp150 stalk-residues 108–151-accounted for half of all the gp150-specific mAbs we derived (Fig. 2F). Overlapping 15-mer peptides covering this region (Fig. 2G) reproduced the cognate epitope of 11/44 mAbs. Thus, the most immunogenic part of gp150 appeared to have a predominantly linear structure. 20/20 gp150-specific mAbs specific for residues 108–269 recognized denatured antigen on immunoblots (data not shown), consistent with the most immunogenic part of the native protein forming linear epitopes.

### Gp150-specific mAbs drive IgG Fc receptor-dependent infection

**Figure 3:**
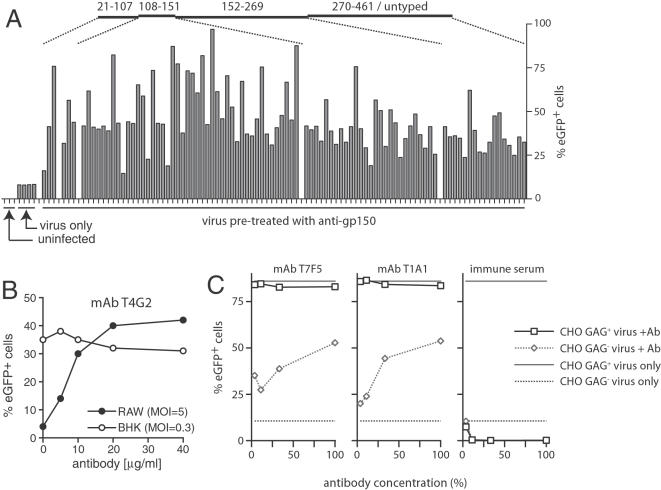
Effect of gp150-specific mAbs on MHV-68 virion infectivity. A. EGFP-expressing MHV-68 virions were incubated with hybridoma supernatants (1 h, 37°C) and then added to RAW264.7 macrophages (3 PFU/cell). 18h later, the number of infected cells was determined by flow cytometric assay of viral eGFP expression. The mAbs are grouped by the region of gp150 recognized. The bars indicate the %eGFP^+^ RAW264.7 cells in each culture. B. EGFP-expressing MHV-68 virions were incubated (1 h, 37°C) with dilutions of the gp150-specific mAb T4G2 and then added to either BHK-21 fibroblasts or RAW264.7 macrophages. 18h later, the number of infected cells was determined by flow cytometric assay of viral eGFP expression. The data shown are representative of 3 equivalent experiments C. EGFP-expressing MHV-68 virions were incubated with gp150-specific mAbs (T7F5, T1A1), with immune serum, or with no antibody and then added to normal, GAG^+^ CHO cells or to the GAG-deficient CHO cell mutant pgs-745 (GAG^−^). Infection was quantitated 18h later by flow cytometric assay of viral eGFP expression.

The immunodominance of gp150 as an antibody target suggested that gp150-specific antibodies might influence significantly the fate of virions exposed to immune sera. We have identified two effects of immune sera on MHV-68 virions: an inhibition of IgG Fc receptor (FcR)-independent infection [24], [37], and an enhancement of FcR-dependent infection [26]. We therefore tested 115 gp150-specific mAbs for their effect on MHV-68 infection of FcR^−^ and FcR^+^ cells, using human cytomegalovirus IE-1 promoter-driven eGFP expression as a readout of infection. This tends to underestimate the total level of infection within a given cell population, but provides a reliable, validated comparison of infection levels between populations [26]. None of the mAbs reduced the infection of BHK-21 cells (FcγR^−^) or NS0 myeloma cells (FcγRII^+^), regardless of whether exogenous mouse complement was present or not (data not shown). All enhanced the infection of RAW264.7 cells (FcγRI^+^ FcγRIII^+^), some to very high levels (Fig. 3A). Gp150-specific mAbs therefore increased virion infectivity (Fig. 3B).

The gp150 positional homolog in EBV-gp350-inhibits EBV infection of epithelial cells [38]. This inhibition can be partly offset by gp350-specific mAbs [39]. Gp150 mediates an analogous inhibition of GAG-deficient cell infection by MHV-68 [27] and gp150-specific mAbs were able to increase MHV-68 infection of GAG-deficient CHO cells (Fig. 3C). However, the enhancement was less marked than for FcR^+^ cells and required more antibody. Also, immune sera contained insufficient gp150-specific antibody relative to infection blocking antibody to enhance GAG-deficient CHO cell infection (Fig. 3C), whereas immune sera do enhance RAW264.7 cell infection [26]. Thus, at least for MHV-68, FcR-independent infection enhancement by gp150-specific antibodies is probably not of great practical importance.

### Most serum-mediated, FcR-dependent infection is due to gp150

**Figure 4:**
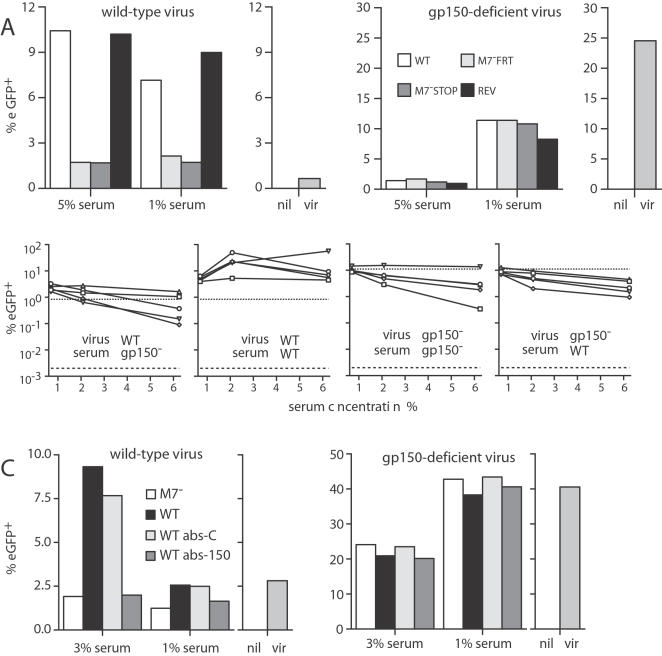
Serum-mediated, FcR-dependent MHV-68 infection depends on gp150. A. Wild-type or gp150-deficient virions were incubated with sera from BALB/c mice, taken 2 months after infection with wild-type (WT), gp150-deficient (M7^-^FRT, M7^-^STOP) or revertant (REV) viruses as indicated. The virus/serum mixtures were then added to RAW264.7 cells (3 PFU/cell). The proportion of infected cells in each culture was determined 18h later by flow cytometric assay of viral eGFP expression. nil = uninfected, vir = virus only. Each serum sample was pooled from 5 mice. The data are from 1 of 2 equivalent experiments. B. Sera were taken from C57BL/6 mice 6 months after infection with either wild-type (WT) or M7^-^STOP (gp150^−^) viruses. EGFP-expressing WT or gp150^−^ viruses were then incubated with sera from individual mice and used to infect RAW264.7 cells. The level of infection was assayed 18h later by flow cytometry of viral eGFP expression. C. Sera were pooled from 5 mice at 6 months after infection with wild-type (WT) or gp150-deficient (M7^-^) MHV-68. Gp150-specific antibodies were removed from wild-type-immune sera by 3 rounds of incubation on 2% paraformaldehyde-fixed CHO-gp150 cells (see Figure 2D) (WT abs-150). Controls were incubated on paraformaldehyde-fixed CHO cells (WT abs-C). Wild-type or gp150-deficient viruses were incubated with each serum sample and then used to infected RAW264.7 cells (3 PFU/cell, 18 h). The data are representative of 2 experiments.

The potency of gp150-specific mAbs in driving FcR-dependent infection suggested that gp150 might be responsible for much of this effect of immune sera. We determined the contribution of gp150 by comparing sera from mice 2 months after infection with either wild-type or gp150-deficient viruses (Fig. 4A). Gp150 knockout-immune sera gave much less enhancement than wild-type. Similar results were obtained at 6 months post-infection (Fig. 4B). Absorbing gp150-specific antibodies out of wild-type-immune serum also largely abolished its infection enhancing effect (Fig. 4C).

**Figure 5:**
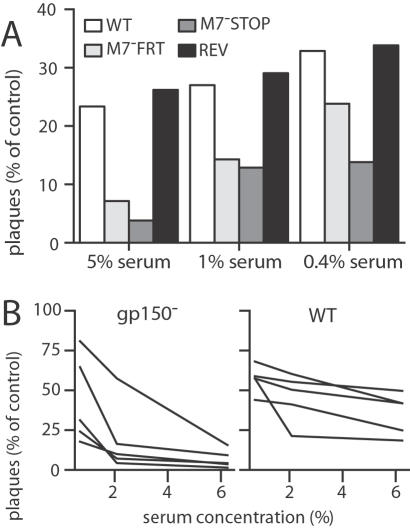
The antibody response to gp150 tends to reduce rather than enhance MHV-68 neutralization. A. Wild-type MHV-68 virions (1000 PFU/sample) were incubated with sera pooled from 5 mice 2 months after infection with gp150^+^ (WT, REV) or gp150^−^ (M7^-^FRT, M7^-^STOP) viruses and then used to infect BHK-21 cells. Control samples were BHK-21 cells infected with virus and no antibody. B. Sera from C57BL/6 mice 3 months after infection with wild-type (WT) or M7^-^STOP (gp150^−^) MHV-68 was used to inhibit wild-type MHV-68 infection of BHK-21 cells. Despite variation between individual mice, plaque titers were significantly lower with the 2% and 6% sera from gp150 knockout-infected mice (p<0.01 by Student's t test).

Removing gp150-specific antibodies from immune sera had no effect on the neutralization of BHK-21 cell infection (data not shown) and pooled gp150 knockout-immune sera neutralized wild-type MHV-68 for BHK-21 cell infection somewhat better than pooled wild-type-immune sera did (Fig. 5A), consistent with gp150 not being a significant neutralization target. More variable but essentially similar results were obtained with individual sera (Fig. 5B). Frequency analysis of glycoprotein-specific hybridomas (n>150) from gp150 knockout virus carriers showed that, except for gp150, the basic immunodominance hierarchy was maintained. The percentages of mAbs specific for each glycoprotein were: gp150-0%, gp70-45%, gB-24%, gH/gL-23%, ORF27-7%, gM/gN-2%. Thus, a lack of gp150 not only had no adverse effect on neutralization, but also allowed greater stimulation of other, potentially neutralizing B cells.

### Boosting gp150-specific immunity boosts IgG Fc receptor-dependent infection

**Figure 6:**
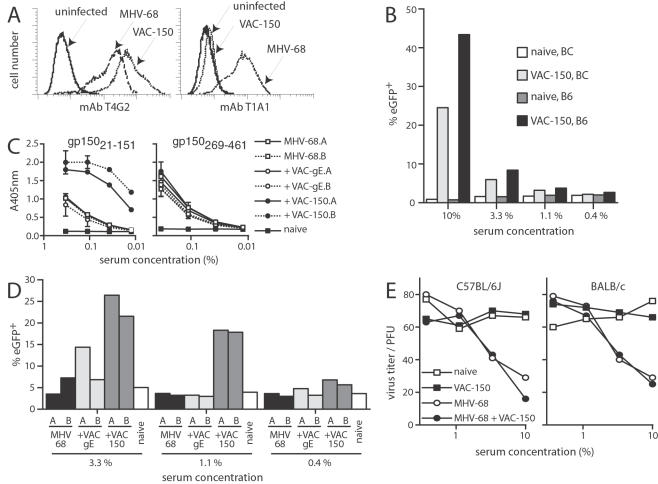
gp150-specific immunity in carrier mice increases FcR-dependent infection and has no effect on neutralization. A. BHK-21 cells were left uninfected (solid lines) or infected (2PFU/cell 18h) with MHV-68 (dashed lines) or VAC-150 (dotted lines), then assayed for cell surface gp150 expression by flow cytometry. MAb T4G2 recognizes an epitope in gp150 amino acid residues 108–151. MAb T1A1 recognizes an epitope in amino acid residues 152–269. VAC-150 expresses residues 1–151 with a GPI anchor. B. BALB/c (BC) or C57BL/6J (B6) mice were either left uninfected or infected with VAC-gp150. At the times post-infection indicated, sera were incubated with eGFP-expressing MHV-68. The serum/virus mixtures were then used to infect RAW264.7 macrophages. Infection was quantitated 18h later by flow cytometric assay of viral eGFP expression. The data are from 1 of 2 equivalent experiments. C. C57BL/6 mice were infected intranasally with MHV-68. 3 months later they were boosted with VAC-150 (+VAC-150) or a control vaccinia virus expressing the Herpes simplex virus gE (+VAC-gE) or left unboosted (MHV-68). Naive = age-matched, uninfected mice. Immune sera were pooled from groups of 3 mice. A and B are separate pools for each treatment arm. The sera were assayed by ELISA for reactivity against N-terminal (residues 21–151) or C-terminal (residues 269–461) fragments of gp150. Only the N-terminal part is expressed by VAC-150. D. The same sera as in C were added to eGFP^+^ MHV-68 virions and the mixtures added to RAW264.7 cells. 18h later, eGFP expression was quantitated by flow cytometry. The data are from 1 of 3 equivalent experiments. E. C57BL/6 and BALB/c mice were either left uninfected (naive), infected with MHV-68 or VAC-gp150, or infected with MHV-68 and then boosted with VAC-gp150. Pooled sera from these mice (5 per group) were then tested for MHV-68 neutralization by plaque assay. Infection with VAC-gp150 made no detectable difference to neutralization titers. The data are from 1 of 2 equivalent experiments.

We also tested whether gp150 mono-specific immune sera could boost FcR-dependent infection. To do this, we generated a vaccinia virus recombinant expressing amino acid residues 1–151 (the most immunogenic region) of gp150 with a GPI anchor (VAC-150) (Fig. 6A). VAC-150 elicited an antibody response from naive mice that increased FcR-dependent MHV-68 infection, whereas a control vaccinia virus recombinant did not (Fig. 6B).

The steady state CD8^+^ T cell response to MHV-68 can be changed by post-exposure vaccination [40]. To test whether the antibody response could be changed in the same way, we super-infected MHV-68 carrier mice with VAC-150. This boosted serum antibody titers to gp150 residues 1–151 without a change in titers to residues 269–461 (Fig. 6C). The boosted sera gave more FcR-dependent infection (Fig. 6D). Immunization with VAC-150 did not, by contrast, affect the neutralization titers of either naive or MHV-68 carrier mice (Fig. 6E). These data confirmed that the gp150-specific component of the antibody response mediates Fc receptor-dependent infection rather than neutralization, and is therefore likely to benefit the virus rather than the host.

## Discussion

Antibody can attenuate herpesvirus-induced disease, but neither prevents viral transmission nor drives obvious viral antigenic variation. Immune sera stop MHV-68 virions binding to fibroblasts. However, they promote FcR-dependent infection [26], suggesting that viral membrane fusion remains intact. Thus, so long as an alternative uptake route is available, antibody-exposed virions remain infectious. Here we analysed the antibody response to the accessible MHV-68 virion surface, in order to establish an immunological basis for FcR-dependent infection. The immunodominant virion antibody target was gp150. Gp150-specific antibodies did not neutralize. Instead, they played the major role in whole sera of driving FcR-dependent infection. The gp150-specific response also suppressed somewhat the neutralizing antibody response. In contrast to gp150, the major mAb-defined MHV-68 neutralization target, gH/gL, was poorly immunogenic. Thus gp150, though its immunogenicity, distorts the antibody response and reduces its impact on virion infectivity. Many complex pathogens could compromise antibody responses with non-essential glycoproteins in the same way.

The most immunogenic region of gp150 lay in its serine/threonine-rich stalk. This appeared to have a linear structure, in that most mAbs recognizing the native protein also recognized denatured forms. The preservation of gp150 epitopes in protein fragments may explain its immunodominance, as infected cell debris-probably a major source of antigen-would continue to stimulate gp150-specific B cells. In contrast, neutralizing epitopes on gH/gL are lost when these proteins dissociate [25], [41]. The simple, predominantly hydrophilic amino acid composition of gp150 could also be important in allowing a range of strong, electrostatic antibody interactions. Despite the diversity of gp150 homologs in different gammaherpesvirus, a serine/threonine rich domain is conserved. The EBV and KSHV homologs-gp350 and K8.1-also appear to be strongly immunogenic [17], [42]. Thus, gp150 immunogenicity could represent a general gammaherpesvirus strategy of antibody subversion. Deliberate immunogenicity might seem counter-productive for EBV, since gp350 contributes to B cell infection [43], but *in vivo* B cell infection may not require cell-free EBV. Direct spread from epithelial cells is also possible.

Gp150 may be specifically adapted to non-standard infection routes. For example, its A/D/E/P/S/T-rich stalk could be sufficiently mobile to accomodate bulky antibodies and still allow membrane apposition for fusion. However, gp150 knockout-immune sera still gave some enhancement of RAW264.7 cell infection (Fig. 4A), as do mAbs specific for other glycoproteins [26], so this effect of gp150 was not unique. Nor, presumably, was its suppression of neutralizing specificities: gp70, second in immunodominance and again non-essential, should have a similar effect. Thus, while gp150 provides a striking example of how immunodominance works against neutralization, other non-essential glycoproteins could have similar effects. How would immunodominance work? Germinal centre B cells must take up and present antigen to survive [44]. Herpesviruses glycoproteins often associate and they all come together in virions. B cells endocytosing one glycoprotein will therefore tend to endocytose others too, much as influenza hemagglutinin-specific B cells also take up non-hemagglutinin antigens [45]. Germinal B cells recognizing different herpesvirus glycoproteins should therefore compete for antigen via their requirement for T cell help, allowing non-neutralizing B cells to suppress neutralizing responses. Notably, the total glycoprotein-specific antibody responses to wild-type and gp150-deficient MHV-68 were very similar (Fig. 1C): when the gp150-specific response was lost, that to other targets increased.

Stewart et al. have argued that gp150 is important for B cell infection [46]. We find no evidence for this. Indeed, gp150-deficient MHV-68 binds to and infects GAG-deficient cells much better than wild-type, not worse [27], [31]. Our data are consistent instead with the glycosaminoglycan binding reported for the gp150 homologs of other gamma-2-herpesviruses [47]–[49]. Gp150 binding to glycosaminoglycans, though weak [31], is still functionally important [27]. Our data suggest that gp150 works not by providing strong binding, but by providing glycosaminoglycan-sensitive regulation of cell binding by another virion glycoprotein, possibly gB [27], [31].

Gp150 mutants show normal host colonization [27]. However, this reflects not that gp150 is unimportant, but that the current MHV-68 pathogenesis model measures mainly primary infection and disease. At least the former is not where gp150 acts. Many MHV-68 lytic genes will contribute to disease, as MHV-68 causes disease mainly by lytic replication [51], [52]. In contrast, EBV and KSHV cause disease mainly when latent. The value of MHV-68 as a specific disease model is therefore debatable, and we have not pursued it. MHV-68 would seem to be a much better model for normal gamma-herpesvirus infection. Primary host colonization by MHV-68 depends more on latency-associated lymphoproliferation than on lytic replication [11]–[13], so here lytic genes that have been conserved over millions of years often have rather minor pathogenesis phenotypes [27], [29], [33], [50]. Defining their true in vivo importance requires a better pathogenesis model. The two known functions of gp150-virion release [27] and antibody evasion-would be consistent with an important role in transmission. MHV-68, like murine cytomegalovirus, is poorly transmitted between conventionally housed mice. Our goal now is therefore to establish experimental transmission by reproducing more accurately the natural social interactions of the infected host.

## Materials and Methods

### Mice and monoclonal antibodies

BALB/c and C57BL/6J mice were purchased from Harlan U.K. Ltd. (Bicester, U.K.), housed in the Cambridge University Department of Pathology and infected intranasally with 3×10^5^ PFU MHV-68 when 6–8 weeks old (Home Office Project Licence 80/1579). Vaccinia viruses were given by intraperitoneal injection (3×10^6^ PFU). For mAb production, mice were boosted with 10^7^ PFU intraperitoneal MHV-68 3–6 months after intranasal infection when both the viral load and serum antibody have reached steady state [24], [37]. Splenocytes were harvested 3 days later and fused with NS0 cells using polyethylene glycol 1500 [53]. Cell hybrids were grown on monolayers of irradiated MRC-5 cells in 20% FCS and selected with azaserine (1 µg/ml)/hypoxanthine (100 µM). MAbs were concentrated from tissue culture supernatant by ammonium sulfate precipitation and quantitated by ELISA using isotype-matched standards.

### Plasmids

The gp150 coding sequence (genomic co-ordinates 69466-70917) [54] was amplified by PCR (Hi-Fidelity PCR kit, Roche Diagnostics Ltd), using 5' *Eco*RI-restricted and 3′ *Xho*I-restricted primers, and cloned into the *Eco*RI/*Xho*I sites of pMSCV-IRES-ZEO [55]. This plasmid was used for retroviral transduction [55] of L929 cells to make a cell line stably expressing gp150. The coding sequence for amino acid residues 1–151 was amplified with 5′ *Xba*I-restricted and 3′ *Not*I-restricted primers and cloned into the *Xba*I/*Not*I sites of pBRAD, thereby attaching a C-terminal glycosyl-phosphatidyl-inositol (GPI) membrane anchor [28]. To fuse gp150 fragments to N-terminal GST, the relevant portions of its coding sequence were amplified by PCR with 5′ *Eco*RI-restricted and 3′ *Xho*I-restricted primers and cloned into the *Eco*RI/*Xho*I sites of pGEX-4T-1 (APBiotech, Little Chalfont, U.K.). Expression in BL-21 *E.coli*. was induced for 4h with 400 µM IPTG. The *E.coli* were lysed in 1% Triton X-100/50 mM TrisCl pH 7.4/150 mM NaCl/5mM EDTA/1mM PMSF/5mM N-ethyl-maleimide/Complete protease inhibitors (Roche Diagnostics). GST fusion proteins were precipitated with glutathione-sepharose beads (APBiotech), washed ×3 in lysis buffer and ×2 in Tris-saline, eluted with 50mM glutathione and dialysed ×3 against PBS.

### Cells

All cells were grown in Dulbecco's modified Eagle medium (Invitrogen, Paisley, U.K.) supplemented with 2mM glutamine, 100U/ml penicillin, 100 µg/ml streptomycin and 10% fetal calf serum (PAA laboratories, Linz, Austria). Where indicated, cells were transfected using Fugene-6 (Roche Diagnostics Ltd., Lewes, U.K.).

### Viruses

MHV-68 was derived from a genomic BAC, which also transcribes eGFP from a human cytomegalovirus IE-1 promoter [56]. For all *in vivo* experiments, the loxP flanked eGFP and BAC sequences were removed by passaging viruses through NIH-3T3-CRE cells [57]. Two, non-overlapping gp150-deficient mutants, M7^-^FRT and M7^-^STOP, and a revertant of the M7^-^FRT mutant have been described [27]. Viruses were grown in BHK-21 cells. Infected cultures were cleared of infected cell debris by low-speed centrifugation (1000×g, 3min). Virions were then concentrated by high speed centrifugation (38000×g, 90min). Virus titers were determined by plaque assay on BHK-21 cells [27]. To make VAC-gp150, the GPI-linked coding sequence for amino acid residues 1–151 was subcloned from pBRAD into pMJ601 [58] and then transfected into vaccinia virus WR-infected TK^-^143 cells. Thymidine kinase-deficient recombinants were selected by passage in TK^-^143 cells with 25 µg/ml 5′-bromo-2′-deoxyuridine (Sigma Chemical Co, Poole, U.K.) and identified by beta galactosidase assay with X-gal. They were purified to homogeneity by limiting dilution cloning.

### Flow cytometry

Cells infected with eGFP^+^ viruses were washed in PBS and analysed directly for green channel fluorescence. For specific staining, cells were incubated with MHV-68 glycoprotein-specific mAbs (1 h, 4°C), washed ×2 in PBS, incubated with fluorescein-conjugated rabbit anti-mouse IgG pAb (Dako Cytomation, Ely, U.K.), washed ×2, and analysed on a FACS Calibur (Becton-Dickinson, Oxford, U.K.). Graphs were plotted with FCSPress v1.3 (www.fcspress.com).

### ELISA

Nunc Maxisorp ELISA plates (Nalge Nunc, N.Y.) were coated (18 h, 4°C) with GST fusion proteins, blocked in PBS/0.1% Tween-20/1% BSA, and incubated with hybridoma supernatant. Bound antibody was detected with horseradish peroxidase-conjugated goat anti-mouse IgG pAb (Sigma). For fine mapping, instead of GST fusion proteins, biotinylated 15-mer peptides (Peptide Therapeutics, Eastleigh, U.K.) were captured onto ELISA plates pre-coated with 10 µg/ml avidin (Sigma). For total MHV-68 recognition, the plates were coated with 0.01% Triton X-100-disrupted MHV-68 virions. The plates were washed ×5 in PBS/0.1% Tween-20 after each step. The detection substrate was nitrophenylphosphatate (Sigma). Absorbance was read at 405nm using a Benchmark ELISA plate reader (BioRad, Hercules, CA).
